# Effect of Tempeh on Gut Microbiota and Anti-Stress Activity in Zebrafish

**DOI:** 10.3390/ijms222312660

**Published:** 2021-11-23

**Authors:** Yo-Chia Chen, Nha-Linh Tao, Shao-Yang Hu, Hui-Yun Tsai, Sin-Chung Liao, Wei-Lun Tsai, Chun-Yi Hu

**Affiliations:** 1 Department of Biological Science and Technology, Pingtung University of Science and Technology, Pingtung 912301, Taiwan; ox@mail.npust.edu.tw (Y.-C.C.); nhalinh2122@gmail.com (N.-L.T.); syhu@mail.npust.edu.tw (S.-Y.H.); 2 Department of Nutrition and Health Science, Fooyin University, Kaohsiung 831301, Taiwan; rhytsai@gmail.com; 3 Department of Biotechnology, Meiho University, Pingtung 912009, Taiwan; a0927608129@gmail.com; 4 Division of Gastroenterology and Hepatology, Department of Internal Medicine, Kaohsiung Veterans General Hospital, Kaohsiung 813414, Taiwan; 5 Department of Food Science and Nutrition, Meiho University, Pingtung 912009, Taiwan

**Keywords:** tempeh, anti-stress, microbiota, BDNF

## Abstract

*Rhizopus oryzae* is a fungus used to ferment tempeh in Indonesia and is generally recognized as safe (GRAS) for human consumption by the USA FDA. We previously assessed the effect of a tempeh extract on cortisol levels in zebrafish but did not include behavioral studies. Here, we measured the GABA content in three strains of *Rhizopus oryzae*, two isolated by us (MHU 001 and MHU 002) and one purchased. We then investigated the effect of tempeh on cortisol and the gut microbiota in a zebrafish experimental model. GABA concentration was the highest in MHU 002 (9.712 ± 0.404 g kg^−1^) followed by our MHU 001 strain and the purchased one. The fish were divided into one control group fed a normal diet and three experimental groups fed soybean tempeh fermented with one of the three strains of *Rhizopus oryzae*. After two weeks, individual fish were subjected to unpredicted chronic stress using the novel tank diving test and the tank light–dark test. Next-generation sequencing was used to analyze gut microbial communities and RT-PCR to analyze the expression of BDNF (brain-derived neurotrophic factor) gene and of other genes involved in serotonin signaling/metabolism in gut and brain. Tempeh-fed zebrafish exhibited increased exploratory behavior (less stress) in both tank tests. They also had significantly reduced gut Proteobacteria (include *E. coli*) (51.90% vs. 84.97%) and significantly increased gut Actinobacteria (include *Bifidobacterium* spp.) (1.80% vs. 0.79%). The content of *Bifidobacterium* *adolescentis*, a “psychobiotic”, increased ten-fold from 0.04% to 0.45%. Tempeh also increases BDNF levels in zebrafish brain. *Rhizopus oryzae* MHU 001 greatly improved the anti-stress effect of tempeh and microbiota composition in zebrafish gut.

## 1. Introduction

Tempeh is a traditional fermented soybean food product consumed mainly in Southeast Asia, especially in Indonesia. It is prepared through fermenting dehulled and cooked soybeans with *Rhizopus* fungi [1]. During fermentation, the fungal mycelium binds the soybeans together into a compact cake [2]. While *R. oligisporous*, *R. oryzae*, and *R. stolonifera* have been identified as soybean tempeh starters, only *R. oligosporous* [3] and *R. oryzae* [4] have been widely used for this purpose. *R. oryzae* is generally recognized as safe (GRAS) by the USA FDA and is commonly used in the preparation of many foods such as sauces and tempeh [5].

Fermentation increases the nutritional value of some nutrients in tempeh. It promotes the release of vitamins, phytochemicals, and antioxidative constituents [6]. Isoflavone levels are higher in tempeh than in other soybean products such as tofu and soy beverages [7]. The fermentation of tempeh also decreases phytic acid and increases the bioavailability of minerals such as calcium, zinc, and iron [6]. Like other soy products, tempeh contains the amino acid GABA [8]. γ-Aminobutyric acid (GABA) is a major inhibitory neurotransmitter in the central nervous system. It prevents the induction of hypertension and diabetes and can have diuretic, sedative, and anti-anxiety effects [9].

There is emerging knowledge regarding the bi-directional crosstalk governing gut-to-brain communication and its relation to health and disease of both organs. While it has been long known that the brain can affect gut functions, the gut has also recently been found to induce changes in the central nervous system (CNS). There is compelling evidence of the various links between the enteric microbiota and brain function [10]. This connection is becoming increasingly relevant to the development of novel therapeutic strategies targeting psychiatric disorders such as depression and anxiety. As such, the absence or modification of enteric microbiota affect stress-associated anxiety-like and depressive-like behaviors [11], leading some to postulate the existence of “psychobiotics,” i.e., live microorganisms that may have beneficial psychotropic effects on their host [12].

For example, some live bacterial strains such as *Lactobacilli* and *Bifidobacteria* can influence CNS function. These bacteria contain several compounds including proteins and peptides as well as cell components that can be potential mediators with the hosts [13]. For example, GABA is a neuroactive molecule generated by psychobiotics and the human gut microbiota that regulates neural signals and thus influences neurological variables as well as sleep, appetite, mood, and cognition [14].

Neurotrophins are proteins involved in neuron development and functioning. BDNF (brain-derived neurotrophic factor) is the best-conserved neurotrophin in vertebrate evolution [15]. As such, the primary amino acid sequences of zebrafish (*Danio rerio*) and human BDNF are 91% identical [16]. The distribution patterns of BDNF mRNA and protein have been described in the CNS of rats, mice, humans, and zebrafish [17], and BDNF has also been observed in other organs and tissues of adult and developing zebrafish [18]. Changes in BDNF signaling have also been found to be relevant to a range of human neuronal and psychiatric disorders as well as biological systems involved in the stress response [19].

Zebrafish are commonly used as an experimental animal model in translational neuroscience and behavioral research [20]. Their behavior easily adapts to new environments, they have a low maintenance cost, and they have a rapid reproductive cycle and produce many offspring. Thus, they are an ideal model species for various studies [21]. Zebrafish exhibit robust behavioral responses to anxiety evoked in novel tank experiments [22] because they have a natural instinct to dive, freeze, and reduce exploration to protect themselves in unfamiliar environments. As they gradually acclimate to new environments, they increase exploration and locomotion, decrease freezing behavior, and increase their entry to the top half of the tank [23].

We previously assessed the effect of a tempeh extract on cortisol, a measure of anxiety, in zebrafish [3]. We did not, however, perform any behavioral studies. Therefore, it is unknown what effect tempeh would have on anxiety-related behaviors. Here, we measured the GABA content of the tempeh extracts and then used zebrafish to assess the effect of tempeh fermented using one of three strains of *Rhizopus oryzae* on stress and the gut microbiota. To do this, zebra fish were divided into four groups: a control group fed a normal diet and three experimental groups, each fed tempeh fermented with one of the three strains. These groups were subjected to two animal behavior studies: a novel tank diving test that measures fish diving depth to assess anxiety as well as a light–dark tank test that measures the time that fish spend in the light to assess anxiety. Next-generation sequencing (NGS) was used to analyze the gut microbiota in the two groups of zebrafish, and RT-PCR was used to measure the central and peripheral expression levels of the BDNF gene—a natural relaxant produced in response to stress—as well as of genes involved in serotonin signaling/metabolism in the brain and gut of the groups of zebrafish [24].

## 2. Result

### 2.1. GABA Detection

**Figure 1:**
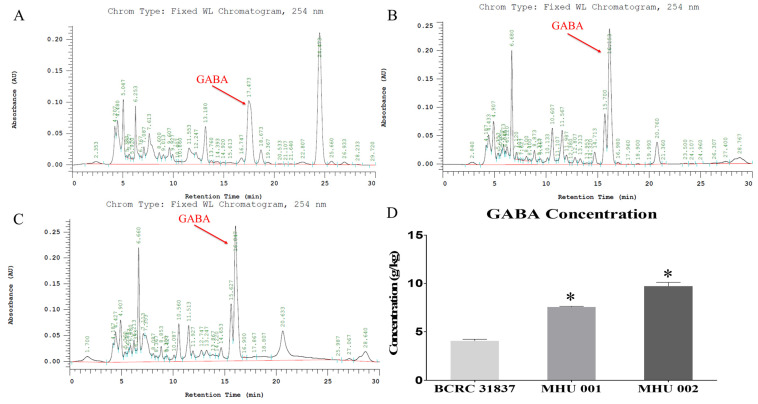
The elution profile of GABA present in tempeh and derivates by high performance liquid chromatography (HPLC) with a C-18 reverse-phase column. Tempeh was fermented by different strains of *R. oryzae*, i.e., BCRC 31837 (**A**), MHU 001 (**B**), and MHU 002 (**C**). GABA concentration of three different kinds of tempeh (**D**). The asterisk indicates a statistically significant difference between the control and the tempeh groups. Bars, means of triplicates ± S.D. (*) *p* < 0.001, as compared with the relative control group by one-way ANOVA.

Six concentrations (0, 200, 400, 600, 800, and 1000 g kg^−1^) were used to optimize the HPLC chromatographic conditions. Figure 1A–D report HPLC data showing the GABA contents in different tempeh samples obtained using the three different strains. Tempeh fermented with *Rhizopus oryzae* MHU 002 had the highest GABA content (9.712 ± 0.404 g kg^−1^) (Figure 1C), followed by *R. oryzae* MHU 001 (7.552 ± 0.081 g kg^−1^) (Figure 1B) and the standard strain *Rhizopus oryzae* (BCRC 31837) (4.122 ± 0.064 g kg^−1^) (Figure 1A).

### 2.2. Novel Tank Diving Test

**Figure 2:**
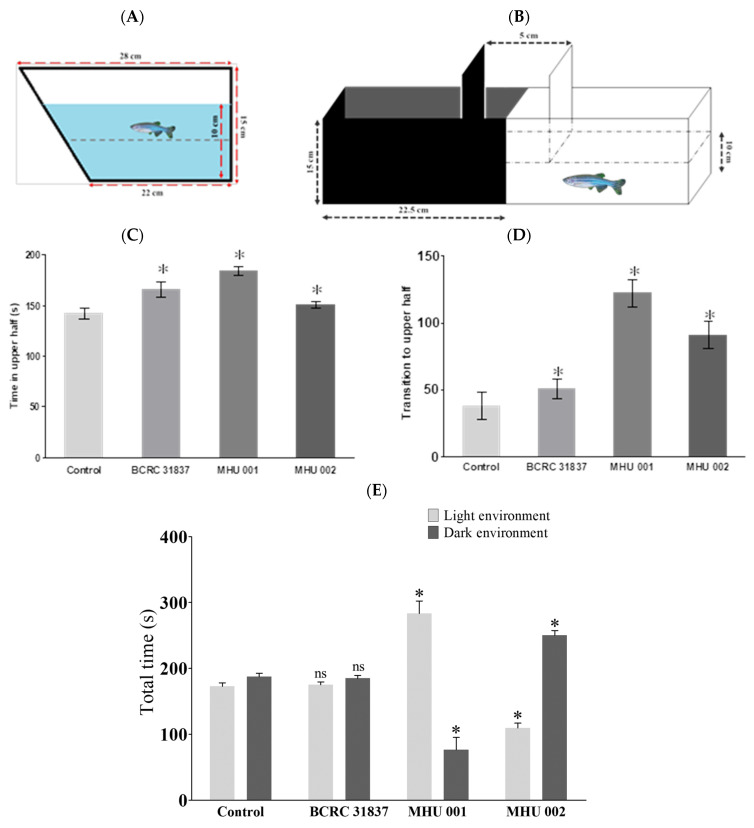
Novel tank diving test for behavioral testing in adult zebrafish. (**A**) Light-dark test tank test for behavioral testing in adult zebrafish (**B**). Novel tank diving test shows the time in seconds that the four groups of adult zebrafish spent in upper part of the novel tank; those in the upper part engaged less anxiously in exploratory behavior. Control groups were fed normal meals, and the experimental groups were fed tempeh fermented with different strains of *R. oryzae* BCRC 31837, MHU 001, and MHU 002. (**C**) Time in the upper half (s); (**D**) transition to the upper half (s), and light–dark tank test (**E**). The asterisks indicate statistically significant differences between the control and the tempeh groups. Bars, means of triplicates ± S.D. (*) *p* < 0.001, (ns) *p* > 0.05, as compared with the relative control group by one-way ANOVA (**C**,**D**) and two-way ANOVA (E), respectively; ns indicates non-significant differences.

In the novel tank diving test, zebrafish that spend less time in the top half of the tank and zebrafish that enter the top half fewer times are considered to be less exploratory, which is an indication that they are experiencing anxiety [25]. The subjects in the three experimental groups (BCRC 31837, MHU 001, and MHU 002) spent more time in the upper half of the tank, indicating greater exploratory behavior and less anxiety than the controls. MHU 001 fish spent almost half of their six minutes there (184.02 ± 4.92 s), followed by BCRC 31837 (165.87 ± 4.97 s), MHU 002 (150.70 ± 1.97 s), and control group (142.056 ± 3.27 s) (Figure 2C). Figure 2D shows that the experimental fish transferred significantly more often from the low to the higher half of the tank with respect to the control group (one-way ANOVA, *p* < 0.05). MHU001 exhibited the greatest number of transitions to the upper half (122.25 ± 6.43 times), followed by MHU002 (91 ± 6.272 times) and BCRC 31837 (51 ± 4.69 times); the control group performed the lowest number of transitions (38.00 ± 6.48 times).

### 2.3. Light–Dark Test

In the light–dark test, zebrafish that spent less time in the light zone of the tank or entered the light zone fewer times were considered to be exhibiting greater exploratory behavior indicating less anxiety. The behavior was recorded by a camera for 6 min (360 s). Figure 2E shows that MHU 001 spent significantly more time in the light zone than the controls (283.47 ± 18.79 s vs. 172.69 ± 5.27 s; two-way ANOVA, (*) *p* < 0.001); MHU 002 spent less time in the lighted zone than the controls (109.67 ± 7.15 s).

### 2.4. Tempeh Modulation of Gut Microbial Communities

**Figure 3:**
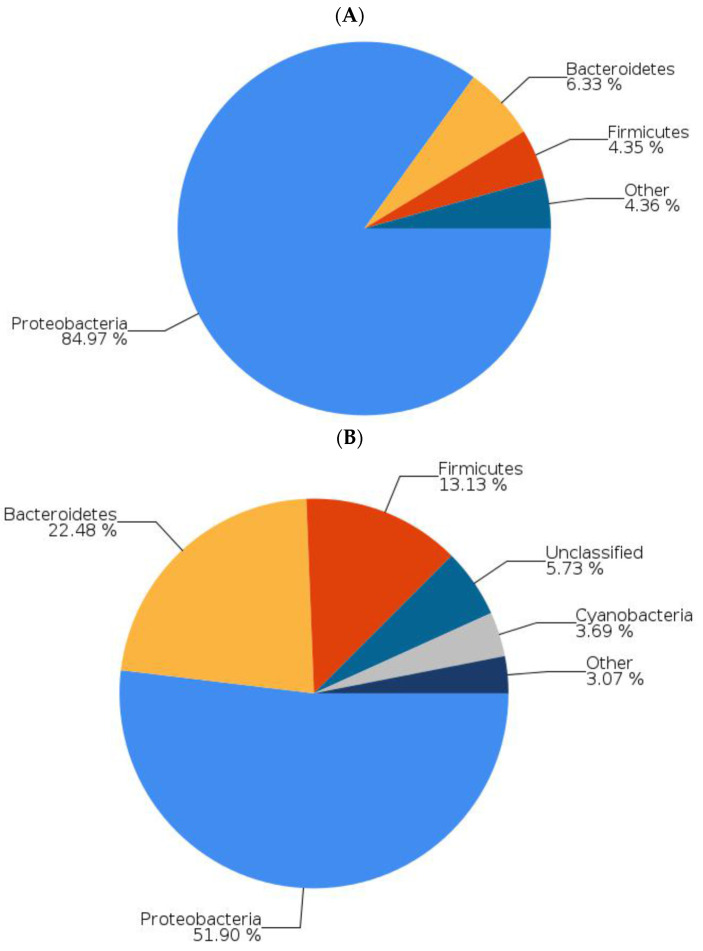
Bacterial structure of different phyla in the gut of zebrafish fed a normal diet (**A**) or fed soybean tempeh fermented with *Rhizopus oryzae* MH-001. (**B**) Data were obtained by next-generation sequencing. Data under 1% not shown.

**Figure 4:**
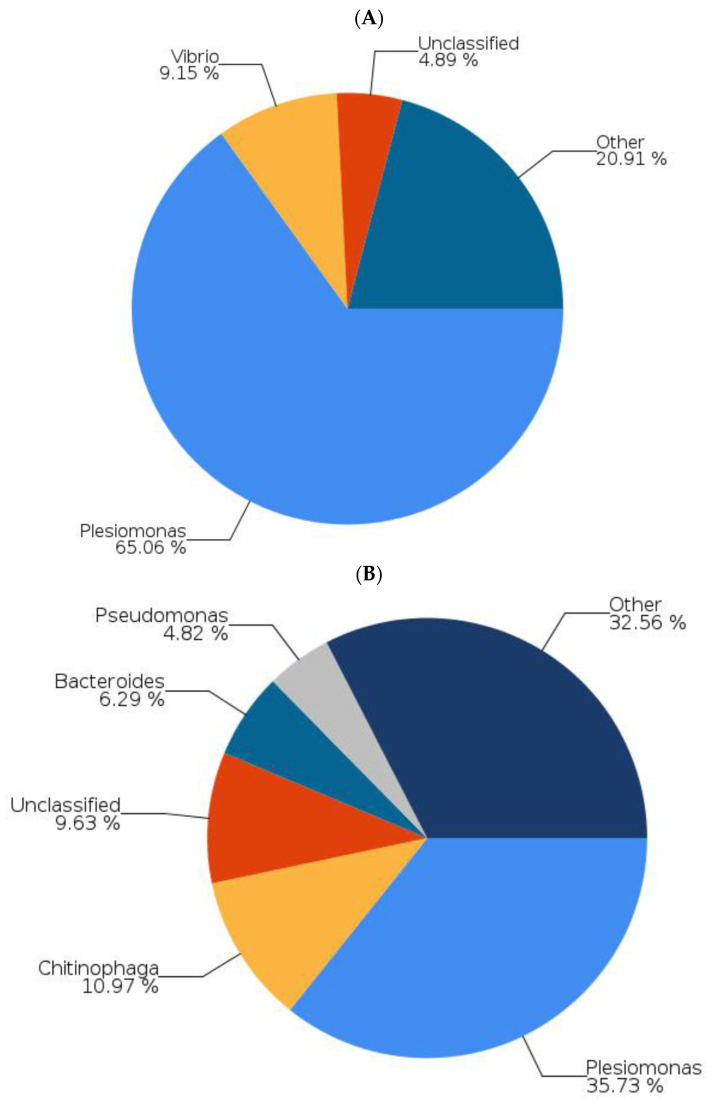
Bacterial structure of different species in the gut of zebrafish fed a normal diet (**A**) or those soybean tempeh fermented with *Rhizopus oryzae* MH-001 (**B**) assessed by next-generation sequencing. Data under 1% not shown.

**Figure 5:**
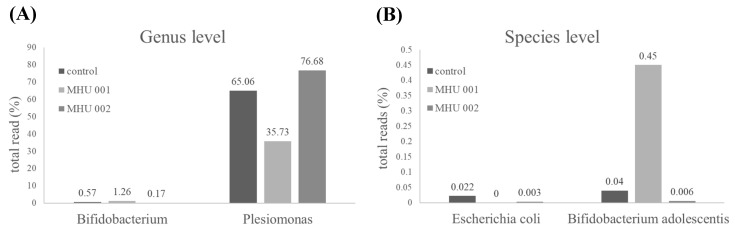
Changes in the distribution of bacteria in the zebrafish gut after feeding soybean tempeh for 2 weeks. Genus level (**A**), and species level (**B**), respectively. Control: normal diet; MHU 001: fed with soybean tempeh fermented with MHU 001; MHU 002: fed with soybean tempeh fermented with MHU 002.

Figure 3 and Figure 4 present data on zebrafish that were not fed tempeh and those fed tempeh for two weeks. Figure 5 shows that Proteobacteria (such as *E. coli*) were reduced from 84.97% (Figure 3A) to 51.90% (Figure 3B) after MHU001 administration while Actinobacteria (such as *Bifidobacterium*) increased more than two times, from 0.79% to 1.80% and from 0.79% to 1.80%. The pie chart in Figure 4 shows the different bacterial species present in the gut of zebrafish fed a normal diet (A) and fed a soybean tempeh fermented with *Rhizopus oryzae* MH-001 (B), assessed by next-generation sequencing. *Plesiomonas* and *Vibrio* are two potentially pathogenic genera belonging to Proteobacteria. These were reduced from 65.06% to 35.73% and from 9.15% to 0.05%, respectively. This change suggests that the administration of tempeh fermented with *Rhizopus oryzae* MH-001 decreased pathogens in the gut. The *Bifidobacterium* genus comprise probiotics and increased from 0.094% to 0.353%.

Figure 5 compares the distribution of important bacteria in the gut of controls and the two tempeh-fed groups: the MHU001 group showed a ten-fold increased content of *B. adolescentis* with respect to either the controls or the MHU002 group (0.45% vs. 0.04% and 0.0006%, respectively). We found a much lower content of *E. coli* in the MHU001 group compared to either the control or the MHU002 group (0.022% vs. 0.0% and 0.003%, respectively). Based on the effect on the distribution of *Bifidobacterium adolescentis* and *E. coli* alone, *Rhizopus oryzae* MHU 001 had a strong beneficial effect on the intestinal flora of zebrafish.

### 2.5. Tempeh Feeding Induces Changes in Neuronal Gene Expression in Brain and Gut

**Figure 6:**
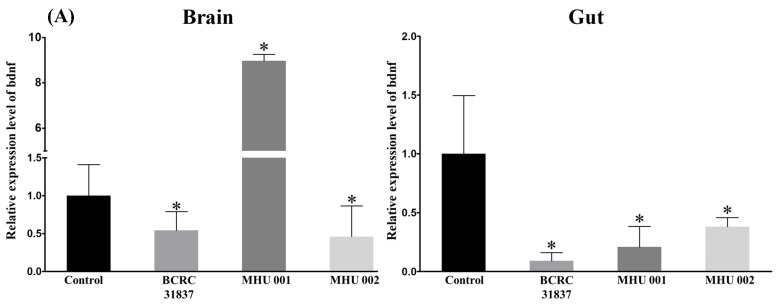

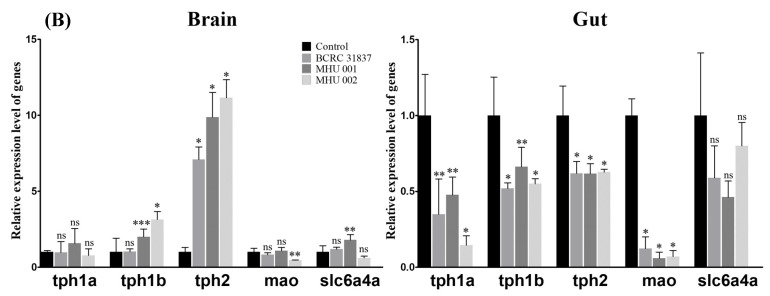
Tempeh feeding induces changes in neural gene expression in zebrafish brain and gut. Controls fed a normal diet. Relative expression of *D. rerio bdnf* (**A**) and *tph1a*, *tph1b*, *tph2*, *htr1aa*, *slc6a4a*, and *mao* (**B**) genes in brain and gut of zebrafish treated with tempeh fermented with different strains of *R. oryzae*, i.e., BCRC 31837, unknown MHU 001, unknown MHU 002, and a control group receiving normal feed. The mean relative expression levels of the tempeh group were normalized to the mean relative expression of the control group. AU, arbitrary units. Bars represent SEM. The asterisks indicate statistically significant differences between the control and the tempeh groups. Bars, means of triplicates ± S.D. (*) *p* < 0.001, (**) *p* < 0.01, (***) *p* < 0.05, (ns) *p* > 0.05, as compared with the relative control group by one-way ANOVA (**A**) and two-way ANOVA (**B**), respectively; ns indicates non-significant differences.

The neuronal gene BDNF is a natural relaxant produced in response to stress and is increased in various brain regions of subjects exposed to social novelty and novel environments. Our goal was to determine if tempeh might have an effect on brain and gut BDNF in zebrafish. As shown in Figure 6, the levels of beta-actin transcripts were stably detected, and the Ct values were 27,835 ± 1.224 and 23.878 ± 0.238, respectively. In the brain, the MHU 001 group presented a higher increase in BDNF compared to the controls (*p* < 0.001) (Figure 6A). In the gut, the BCRC 31837, MHU 001, and MHU 002 groups showed significant reductions in BDNF compared to the controls (*p* < 0.001) (Figure 6A). Socio-emotional behavior in vertebrates is heavily dependent on the serotonergic system [26]. Moreover, microbial influence on the serotonergic system may be a key mediator of gut–brain signaling [27]. Therefore, we next investigated potential changes in the expression of selected genes involved in serotonin signaling and metabolism (*tph1a*, *tph1b*, *tph2*, *htr1aa*, *slc6a4a*, and *mao*) in the gut and in the brain of zebrafish fed with one of the three kinds of tempeh (Figure 6B). In the brain, there was a significant increase in the expression of *tph1b* (MHU 001, *p* < 0.05; MHU 002, *p* < 0.001), *tph2* (BCRC 31837, MHU001, 002, *p* < 0.001), and *slc6a4a* genes (MHU 001, *p* < 0.01), suggesting that feeding with tempeh changed the expression levels of genes involved in the serotonergic system. In contrast, in the gut, we noted these genes expression reduced, with significant differences in the expression levels of all the five genes, except *slc6a4a* gene. These findings collectively suggest tissue-specific modulation of the serotonergic system (Figure 6B).

## 3. Discussion

Zebrafish is an increasingly important model organism in neuroscientific basic and translational research [28]. The digestive tract of zebrafish is similar to that of mammals in its development, organization, and function. Zebrafish is well suited for studying host–microbe interactions because they have innate and adaptive immune systems similar to those of higher vertebrates [29]. Here, we used a zebrafish model to assess the effect of tempeh fermented with three different strains of *R. oryzae* on anxiety-related behavior in fish tank tests, microbial communities in the gut, and one biomarker of the stress response in brain and gut. Our tank tests showed that tempeh-fed fish exhibited greater exploratory behavior and less anxiety than control fish. All three groups of tempeh-fed fish (BCRC 31837, MHU 001, and MHU 002) spent significantly more time in the upper half of the tank in the diving test (*p*-value; Figure 2C). Two groups of fish fed tempeh also spent more time in the light zone of the tank (*p* < 0.05; Figure 2E). Tempeh-fed zebrafish showed a significant decrease in Proteobacteria (e.g., *E. coli* 51.90%) and greater increases in Actinobacteria (e.g., *Bifidobacterium* 1.80% and *B adolescentis)* (Figure 5B). Tempeh-fed fish also exhibited higher levels of BDNF and of several genes (*tph1a*, *tph1b*, *tph2*, *mao*, *slc6a4a*) involved in serotonin signaling metabolism in the brain and lower levels of these neuronal genes in the gut (*p*-values Figure 6). These results reveal previously undescribed behavioral and neurochemical changes in healthy zebrafish induced by an alteration of the enteric microbiota by tempeh. Brain-Derived Neurotrophic Factor (BDNF) is one of the best-conserved genes throughout vertebrate evolution. The BDNF hypothesis of depression postulates that a loss of BDNF is directly involved in the pathophysiology of depression and that its restoration may underlie the therapeutic efficacy of antidepressant treatments [30]. The other tested genes are also involved in intestinal serotonin signaling: *slc6a4*, involved in serotonin transport, *tph1*, associated with the biosynthesis of serotonin, as *tph* encodes tryptophan hydroxylase, *mao*, associated with serotonin metabolism (Zebrafish also have one functional monoamine oxidase gene (*mao*) exhibiting a strong affinity profile for serotonin) [31,32].

We found that tempeh-fed zebrafish exhibited greater exploratory behavior and less anxiety than control fish. They had much lower levels of *E. coli* and much higher levels of *Bifidobacterium* (especially *B. adolescentis*) in the gut as well as higher levels of BDNF in the brain and lower levels of this neurotrophin in the gut. Host–microbes interactions in the gut are now widely thought to affect brain function and behavior and are a potential target in psychiatric diseases [33]. BDNF can across the blood–brain barrier and is an important neurotrophic factor in the brain [34]. It can strengthen neuroplasticity, participate in the growth and differentiation of nerves, and is closely related to cognitive functions such as brain memory and resilience [24]. The microbiota can increase the concentration of BDNF in the hippocampal gyrus of the brain where it has been related to memory and learning as well as to exploratory behavior in animals [35]. Recent studies have also associated BDNF with behavior similar to that elicited by anti-anxiety drugs and antidepressants [24]. The strain 001 group induced a higher increase in BDNF gene expression in zebrafish brain than strain 002 and BCRC 81387, which revealed that 001 tempeh had an effect similar to that of anti-anxiety chemicals and antidepressants. Strain 002 and BCRC 81387 caused a reduction of BDNF and serotonin-related genes, as shown in Figure 6, which reflects the results of behavior experiments (Figure 2C–E) and the gut microbiota analysis (Figure 5). This suggests that the strain 002 and BCRC 81387 are not good candidates for eliciting anti-stress and anti-depression effects.

Our result in Figure 1D, Figure 2E and Figure 6A revealed that GABA concentration is not the main factor involved in the anti-stress activity. We found that tempeh fermented with *Rhizopus oryzae* MHU 002 had the highest GABA content (9.712 ± 0.404 g kg^−1^, Figure 1D); however, MHU 002-fed fish spent less time in the light zone than the controls (109.67 ± 7.15 s, Figure 2E); moreover, the MHU 002 group had the lowest increase in BDNF in the brain (Figure 6A). Therefore, strain 002 presented the highest GABA production compared to other strains, but had a negative effect on stress-like behaviors, gene expression, and the composition of the microbial community, suggesting that other components in tempeh may also affect the stress response. Chen et al. [3] used a red bean tempeh extract in zebrafish experiments to significantly reduce cortisol in zebrafish. In that study, one strain with lower GABA content than the other strains actually had a better effect on cortisol concentration, indicating that other components in red bean tempeh may also affect stress-related cortisol. Here, Proteobacteria (e.g., *E. coli*) were reduced from 84.97% to 51.90% in zebrafish fed tempeh fermented with MHU001 for 2 weeks, while Actinobacteria (e.g., *Bifidobacterium*) increased more than two times from 0.79% to 1.80%. Among Actinobacteria, *Bifidobacterium adolescentis* made up 0.45% in the MHU001 group, but was only 0.04% and 0.006% in the controls and in fish fed MHU002, respectively. That change constitutes a ten-fold increase in *B. adolescentis* in fish fed MHU001.

Duranti et al. conducted a genetic analysis of more than 1000 publicly recorded *Bifidobacterium* strains and found that *Bifidobacterium adolescentis* is a typical GABA producer in the human gastrointestinal tract [36]. Their screening of human/animal total genomic data also revealed a correlation between the amount of *Bifidobacterium adolescentis* and mental burden and disorders including depression and anxiety. When screening 82 strains of *Bifidobacterium adolescentis*, they found two high GABA-producing bacteria, BPRL2019 and HD17T2H, and used them to conduct experiments in rats. Groningen rats fed these *Bifidobacterium adolescentis* strains had higher levels of GABA, highlighting the significance of the potential action of these bacteria on the intestinal–brain axis [36].

This study’s analysis of the microbiota composition found that Proteobacteria, Bacteroidetes, and Firmicutes appeared consistently in the gut microbiota of zebrafish (Figure 5A). Proteobacteria and Fusobacteria are common members of the gut microbiota in adult zebrafish and are especially well adapted to conditions of the fish gastro-intestinal tract in a surrounding aquatic environment [37]. Here, zebrafish fed MHU001 had a significantly higher percentage of Firmicutes (including *Streptococcus* spp. and *Lactobacillus* spp.) than the group not fed tempeh (13.13% vs. 4.35%); they also showed a much lower concentration of Proteobacteria (including *Vibrio* spp. and *Plesiomonas* spp.) than the controls (51.90% vs. 84.97%) (Figure 5). This result is in agreement with previous studies reporting increased abundance of *Lactobacillus* and *Streptococcus* in zebrafish after probiotic administration, resulting in several beneficial effects [38]. Interestingly, a higher proportion of Proteobacteria, together with a lower abundance of Firmicutes, characterizes the intestinal microbiota dysbiosis in a zebrafish model of the inflammatory bowel disease (IBD)-like colitis and correlates significantly with enterocolitis severity, mirroring changes in the human gut microbiota in IBD [39].

This study does have some limitations. One limitation is that we did not measure isoflavones. Another limitation is that we only used three strains of bacteria in this study, and construction of a phylogenic tree are necessary to confirm the effects of the new strain (Figure S1). Moreover, additional animal behavior experiments, such as the freezing test, the predator avoidance test, and the shoaling test, will provide useful data about the anti-stress activity of tempeh [40]. We only evaluated alpha- and beta-diversity when analyzing the microbiota. More strains could be used in the future.

## 4. Materials and Methods

### 4.1. Microorganisms

Standard fungi *Rhizopus oryzae* (BCRC 31837) were obtained from the Bioresource Collection Research Center (BCRC) at the Food Industry Research and Development Institute (Hsinchu, Taiwan). Indigenous *Rhizopus* (*Rhizopus oryzae* MHU 001 and MHU 002) were screened from rotten fruit peels. *Rhizopus* strains were cultured in a potato dextrose agar (PDA) medium and incubated at 37 °C for 2 to 5 days.

For *Rhizopus* activation, 45 g of malt extract powder (Oxoid Ltd., Basingstoke, UK) was mixed with one liter of reverse-osmosis water. It was then boiled with stirring for one minute to completely dissolve the powder. After sterilization at 120 °C for 20 min, the mixture was poured into a Petri dish and test tube and cultured in potato dextrose agar (Lab M Ltd., Heywood, UK) slant medium at 5 °C for 5 to 7 days. Growth was observed with a microscope. The supernatant of the spores of the mycelium (approximately 1 × 10^6^/mL) was filtered through four layers of sterilized gauze (Belia, Jiangxi province, China), and the spore yield was calculated.

### 4.2. Tempeh Fermentation

Soybeans were washed and soaked for 12 h. After drying, the soybeans were treated with water (twice the weight of the soybeans) and 1% lactic acid and cooked at 100 °C for 30 min. The cooked beans were then dried and cooled to 37 °C. The beans (100 g) were then placed on an aluminum plate (10 × 20 cm) with an aluminum cover and thoroughly mixed with 5 mL of *Rhizopus oryzae* containing 1 × 10^6^/mL spores. The mixture was incubated for 2 days in an aerobic environment at 37 °C. The fermented tempeh product was then freeze-dried for 2 days and ground to a powder for analysis.

### 4.3. Aminobutyric Acid Determination

Samples were prepared according to a previously described method with some modifications (Chen et al., 2020): 20 μL of the supernatant was blow-dried with nitrogen. Next, 40 μL ethanol/water/triethylamine (2:1:1) was added for derivatization, and the mixture was blow-dried again with nitrogen. Then, 60 μL ethanol/water/triethylamine/ethyl isothioate (7:1:1:1) was added and allowed to react for 20 min before drying with nitrogen; 20 μL of the mobile phase was then dissolved, filtered through a 0.45 μm filter, and analyzed by HPLC (Hitachi Co., Tokyo, Japan). The HPLC conditions were as follows: mobile phase A/B = 80:20; A: 8.205 g of nitric acid (CH_3_COONa), 0.5 mL of triethylamine, 0.7 mL of acetic acid, and 5 mL of acetonitrile, pH 5.8; B: acetonitrile: water = 60:40, pH 5.8; flow rate: 0.6 mL/min; absorbance: 254 nm).

### 4.4. Animals and Housing

Adult wild-typed zebrafish (*Danio rerio*, strain WIK) [41] were purchased from Taiwan Zebrafish Core Facility (TZCF), Miaoli, Taiwan. All fish were maintained in a circulating system 12:12 light–dark cycle, with two feedings per day, and constant aeration. The internal environments of the tanks were maintained at a temperature of 25 ± 2 °C. After the animals were acclimated for one week, they were divided into four groups (n = 30 per group): (1) a blank group, fed normal food, (2) a control group, fed a mixture of normal food and tempeh fermented by *R. oryzae* (BCRC 31837), (3) the MHU 001 group, fed a mixture of normal food and tempeh fermented using one unknown strain, and (4) the MHU 002 group, fed a mixture of normal food and tempeh fermented using another unknown strain. All fish were fed two times a day for two weeks during the behavioral experiments.

### 4.5. Behavioral Testing

#### 4.5.1. Novel Tank Diving Test

The novel tank diving test records and analyzes fish diving behavior. Anxious fish dive to the lower level of a tank for protection. Less anxious fish spend more time in the upper level. This experiment was performed in a trapezoidal tank (15 cm height × 28 cm top × 22 cm bottom × 10 cm width), shown in Figure 2A. The tank was divided into two equal virtual horizontal portions marked by a dividing line on the outside wall. The novel tank was positioned to help record the behavior from the wide side of the tank using a camera placed on a tripod. One fish was placed in the novel tank. After a 1 min acclimation, its swimming behavior was recorded by the camera over a 6 min period. The behavior was analyzed following the procedures outlined previously [42].

#### 4.5.2. Light–Dark Test

A light–dark test is designed to test anxiety levels. Subjects that stay in the dark are considered to be anxious and seeking protection from darkness. Less anxious subjects move to the light areas to explore new territory. Here, a rectangular tank (15 cm × 10 cm × 45 cm height × width × length) was divided equally into a portion with black walls and a portion with white walls, and the bottom of the tank was either black or white, with only one side of the tank left open for observation [43], as shown in Figure 2B. An individual fish were placed in the test tank at a water depth of 10 cm. A camera was used for 6 min to record three behaviors: immobility duration (s), number of entries into the light zone, and total time spent in the light zone (min).

#### 4.5.3. Euthanasia

All fish were euthanized by rapid freezing in ice. The brain and gut were immediately collected, and stored at −80 °C

### 4.6. Gut Microbiotal Analysis

#### 4.6.1. Fecal DNA Extraction

Genomic DNA was extracted from the fecal samples with the QIAmp DNA Stool Mini Kit (Qiagen, Hilden, Germany) according to the manufacturer’s instructions. The concentration of DNA was assessed by NanoDrop 2000 Spectrophotometer.

#### 4.6.2. PCR Amplification and 16S Sequencing

A library was constructed with the standard V3–V4 region of the 16S rRNA gene. PCR was performed with the KAPA HiFi Hotstart Readymix (Roche, Basel, Switzerland) following the instructions for Illumina 16S Metagenomics Sequencing Library preparation [44]. The PCR products were further purified with AMPure XP magnetic beads (Beckman Coulter, Brea, CA, USA) and barcoded using the Nextera XT Index Kit (Illumina, San Diego, CA, USA). The amplification and quality of PCR products were assessed using a Fragment Analyzer (Advanced Analytical, Ankeny, IA, USA) and quantified by Qubit dsDNA HS Assay Kit (Life Technologies, Carlsbad, CA, USA). The library was then sequenced on a MiSeq (Illumina, San Diego, CA, USA) with paired-end reads (2 × 300 bp) using a MiSeq Reagent Kit V3600 cycles including a minimum of 100,000 reads per sample.

#### 4.6.3. Bioinformatics Analysis

The raw paired-end reads were trimmed, and those that passed the quality filters were assigned to operational taxonomic units (OTU) with a ≥97% similarity as those in the GreenGene Database. The raw paired-end reads were also analyzed using a BaseSpace RDP classifier (Illumina, USA). OTU taxonomic (relative abundance, heatmap), alpha diversity (Shannon index, Venn diagram), and beta diversity (PCoA) were determined using BaseSpace (Illumina, USA), CLC genomics workbench (Qiagen, Germany) and GraphPad Prism 7 (GraphPad Software, San Diego, CA, USA). A *p*-value less than 0.05 was considered significant.

### 4.7. RNA Extraction and Expression Analysis

#### 4.7.1. RNA Extraction

The samples were incubated with 1 mL of TRIzol (Thermo Fisher Scientific Inc., Waltham, MA, USA) and placed at room temperature for 5 min. Then, we added 200 µL of chloroform, shook them for 15 s, and placed them in an icebox for 10 min. The upper layer was collected after centrifugation for 15 min at 4 °C and 12,000 rpm. Then 500 µL of isopropanol was added, and the samples were placed at −20 °C for 1 h in a stainless-steel chamber. The samples were then centrifuged at 12,000 rpm, 4 °C, for 10 min, and the supernatant was removed. The pellets were washed with 1 mL of ethanol 75% and then centrifuged again (8000 rpm, 4 °C, 5 min). After addition of 50 µL of DEPC water, the samples were placed in a stainless-steel chamber at 50–60 °C for 10 min to dissolve the RNA pellet. RNA samples were maintained in a freezer (−80 °C).

#### 4.7.2. cDNA Synthesis

The RNA samples were quantified using a Nanodrop ND-2000 Spectrophotometer (Thermo Scientific, Waltham, MA, USA). These samples were reverse-transcribed using iScript™ cDNA Synthesis kit (Bio-Rad, Hercules, CA, USA). The reaction conditions were set as follows: 25 °C for 5 min, 42 °C for 30 min, 85 °C for 5 min, and 4 °C indefinitely. The cDNA samples were maintained in a freezer (−20 °C).

#### 4.7.3. Real-Time Quantitative Polymerase Chain Reaction (qPCR)

Expression levels of BDNF in the gut and brain were evaluated by RT-PCR. The composition of each reaction sample is shown in Table 1, and the primer design is described in Table 2. The reactions were conducted in technical triplicates and biological duplicates. They were run in an Applied Biosystems Step One Plus Real-time PCR system using the following thermal cycle: 50 °C, 2 min; 95 °C, 10 min; 40 cycles of 95 °C, 15 s, and 60 °C, 1 min, followed by a melting curve cycle. The mean relative expression ratio of the target genes was calculated using the β-actin gene as an endogenous control, applying a previously reported formula [45], and the 2^−∆∆Ct^ formula was used to calculate gene expression difference between the control group and the experimental group.

### 4.8. Statistical Analysis

Data were analyzed by one-way or two-way analysis of variance using GraphPad Prism 9.0 software (GraphPad Software, San Diego, CA, USA). Asterisks indicate statistically significant differences between the control and the experimental groups. Bars, means of triplicates ± S.D. (*) *p* < 0.001, (**) *p* < 0.01, (***) *p* < 0.05, (ns) *p* > 0.05, as compared with the relative control group; ns indicates non-significant difference.

## 5. Conclusions

In conclusion, *Rhizopus oryzae*-fermented soybean tempeh improved the gut microbiota composition and *BDNF* expression in zebrafish in pre-stress and post-stress conditions. Fermentation with *R. oryzae* MHU 001 improved the tempeh’s beneficial effect on stress, as evidenced by a reduction in stress-like behaviors and changes in the expression of *BDNF* and *slc6a4a* and in the structure of the microbial community. For example, *Bifidobacterium* species showed a ten-fold increase in *Bifidobacterium adolescentis* in the gut of zebrafish. MHU 002 fermentation led to the highest GABA content and the highest *tph1b* and *tph2* gene expression. Though strain 001 had less GABA than strain 002, it produced the best anti-stress effect as shown by the results of behavioral tests as well as of gene expression and microbial community analyses, suggesting that other components in tempeh may also affect the stress response. Our new strains greatly improved the anti-stress effect of tempeh and the structure of the microbiota in zebrafish gut. We will determine the sequence of the new strains, build their phylogenic tree, and develop more methods to understand their anti-stress activity in the future.

## Figures and Tables

**Table 1:** Composition of the reaction mixture.

Composition	Volume (µL)
RNA sample (1 µg/µL)	1 µL
5× iScript Reaction Mix	4 µL
iScript Reverse Transcriptase	1 µL
Nuclear-free water	To 20 µL

**Table 2:** Sequence (5′–3′) of the primers.

Gene	GenBank Accession Number	Forward	Reverse
*β-actin*	NM_131031	CACAGATCATGTTCGAGACC	GGTCAGGATCTTCATCAGGT
*bdnf*	U42489	GCTCAGTCATGGGAGTCC	ATAGTAACGAACAGGATGG

## Supplementary Materials

The following are available online at https://www.mdpi.com/article/10.3390/ijms222312660/s1.
