# Supplementary material for: Effect of Tempeh on Gut Microbiota and Anti-Stress Activity in Zebrafish
Source: Int J Mol Sci. 2021 Nov 23;22(23):12660. doi: 10.3390/ijms222312660 (PMC8658004; doi:10.3390/ijms222312660)
Supplement: Supplementary file 1 [file ijms-22-12660-s001.zip › ijms-1381126-supplementary.pdf]

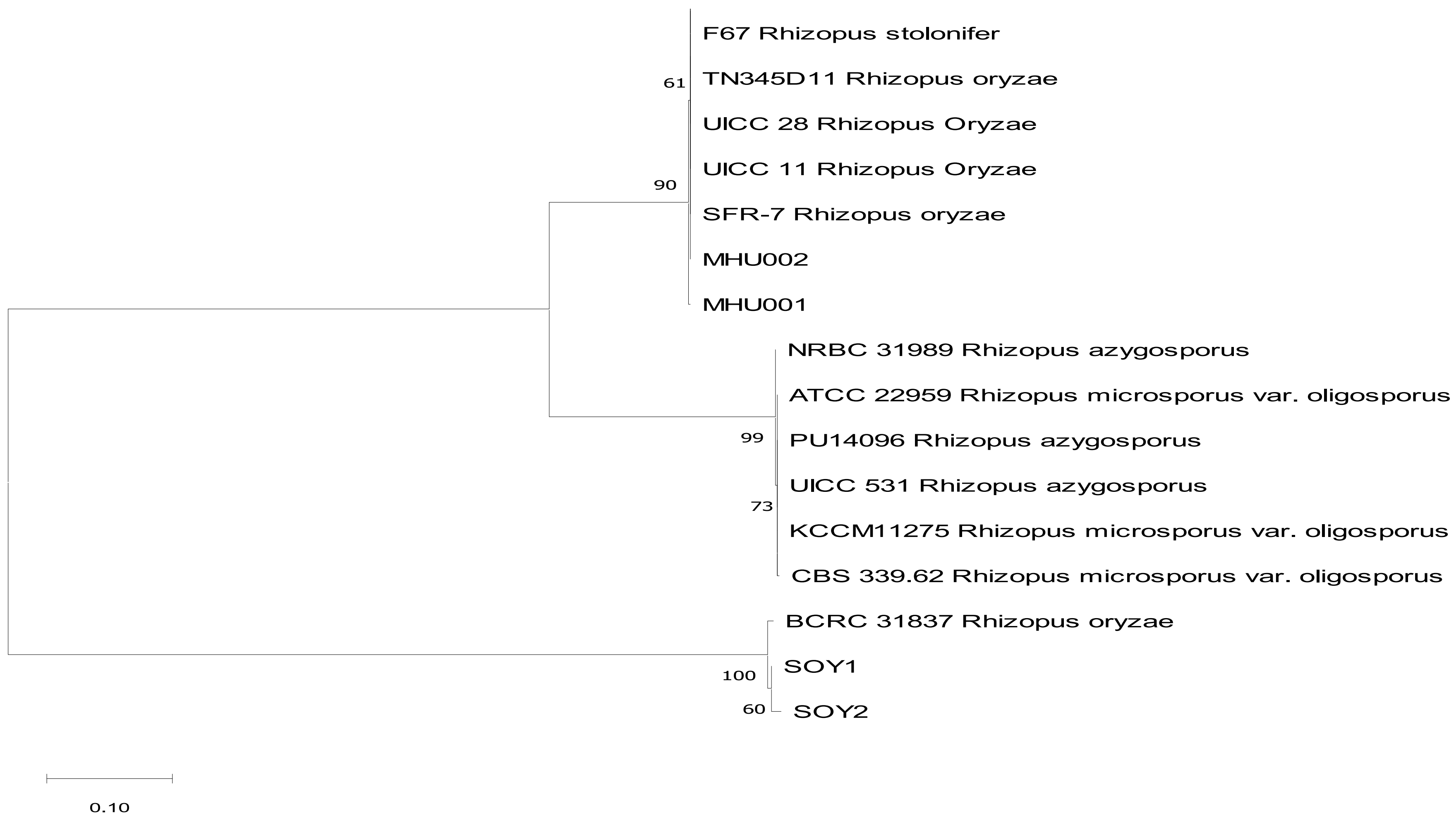

Figure S1. Phylogenetic tree using internal transcribed spacer (ITS) sequences showing closest known relatives of *Rhizopus oryzae*. DNA sequences from the NCBI nucleotide database were aligned using ClustalW, and a phylogenetic tree was constructed using the neighbor-joining (ML) method implemented in MEGA X. The numbers at the branch points are bootstrap values for parsimony-based analysis.
